# Incorporating Forced Migration and Alternative Ontologies into Life Course Theory: Insights from Hmong Refugees

**DOI:** 10.1093/geroni/igaf122.4144

**Published:** 2025-12-31

**Authors:** Michal Engelman, Maichou Lor

**Affiliations:** University of Wisconsin-Madison, Madison, Wisconsin, United States; University of Wisconsin-Madison, Madison, Wisconsin, United States

## Abstract

Hmong refugees were resettled in Wisconsin after the Secret War in Laos. Their life experiences and unique ontology offer new insights into the intersection of forced migration and life course studies. Hmoob Lub Neej (Hmong People’s Lives) is a community-engaged study of aging in Wisconsin’s Hmong refugee community. We conducted semi-structured in-depth life history interviews with 55 older Hmong men and women. Interviews in two Hmong dialects were transcribed and translated into English. Using the five principles of life course theory (history and geography; trajectories and transitions; timing; agency; linked lives), we analyze the disruption and reconstitution of Hmong lives and communities. Participants describe the intersection of history, geography, and biography as they experienced violence and forced relocations. They also address the reconstruction of their livelihoods and communities, highlighting agency and linked lives. The narratives tie early and midlife hardships with later-life outcomes, and showcase social ties – and their absence – as sources of both tension and resilience. In addition, Hmong ontology offers a set of culturally and historically-specific ideas of time, place, spirits, health and life that together generate important new considerations for understanding disruptions to individual and collective life courses. Longing for a spiritually meaningful yet unreachable home and rapid social change in the diaspora define this Hmong cohort’s experience of aging. Their story connects the universal experience of aging and intergenerational change to themes of displacement and inequality, and to diverse ontologies of the life course.

